# The impact of radioligand therapy on prognosis in patients with lung neuroendocrine tumors

**DOI:** 10.3389/fendo.2026.1738286

**Published:** 2026-01-26

**Authors:** Katarzyna Jóźwik-Plebanek, Marek Saracyn, Adam Daniel Durma, Maciej Kołodziej, Weronika Mądra, Mirosław Dziuk, Katarzyna Janiak, Zuzanna Balcerska, Katarzyna Gniadek-Olejniczak, Grzegorz Kamiński

**Affiliations:** 1 Department of Endocrinology and Isotope Therapy, Military Institute of Medicine – National Research Institute, Warsaw, Poland; 2 Faculty of Medicine, University of Warsaw, Warsaw, Poland; 3 Nuclear Medicine Department, Military Institute of Medicine - National Research Institute, Warsaw, Poland; 4 Neurorehabilitation Clinic, Military Institute of Medicine - National Research Institute, Warsaw, Poland

**Keywords:** 177-Lu, 90-Y, lung neuroendocrine neoplasms, lung neuroendocrine tumors, NEN, NET, outcomes, RLT

## Abstract

**Background:**

Evidence on the efficacy and safety of radioligand therapy (RLT) in lung neuroendocrine tumors (LNETs) remains scarce. The limited data available, derived mainly from retrospective analyses are based on small patient cohorts and heterogeneous treatment protocols. The objective of this study was to assess the efficacy and safety of RLT in patients with SSTR–positive LNETs treated with either [¹^77^Lu]Lu-DOTA-TATE or tandem therapy with [^90^Y]Y-DOTA-TATE/[¹^77^Lu]Lu-DOTA-TATE at Polish ENETS Center of Excellence.

**Methods:**

We conducted a retrospective analysis of 22 LNET patients who received RLT and had complete follow-up data. Treatment response and survival outcomes were evaluated. Progression-free survival (PFS) and overall survival (OS) were estimated using the Kaplan–Meier method. Prognostic associations with PFS and OS were explored using univariate and multivariable Cox proportional hazards models, treatment-related AE were graded according to CTCAE.

**Results:**

A total of 22 patients with LNETs (Med. 61 years; 68.2% male) were included. Histology comprised 31.8% typical carcinoid, 54.5% atypical carcinoid, and 13.6% LNET G3. 14 patients received [¹^77^Lu]Lu-DOTA-TATE and 8 tandem [^90^Y]Y/[¹^77^Lu]Lu-DOTA-TATE. At a median follow-up of 54 months, median PFS and OS were 16.0 months (95% CI: 11.2–20.8) and 62.0 months (95% CI: 30.7–93.3), respectively. PFS was longer in patients with high SSTR uptake (34vs16 months; p=0.021) and, in unadjusted exploratory analyses, in those treated with tandem therapy (34vs16 months; p=0.037). OS differed significantly by histology and by prior chemotherapy, while FDG-avid disease was associated with shorter PFS and OS. However, these subgroup comparisons are based on a very small sample and should be regarded as exploratory and interpreted with caution. Treatment was generally well tolerated, with hematologic toxicity being the most common.

**Conclusions:**

RLT demonstrated signals of clinically meaningful activity and an acceptable safety profile in patients with advanced LNETs in this small retrospective cohort. Outcomes were numerically more favorable in individuals with high SSTR uptake and in those treated with tandem therapy, but the study was not designed to compare treatment regimens. These exploratory findings should be regarded as hypothesis-generating only and do not provide evidence of comparative efficacy.

## 1 Introduction

Lung (also referred to as bronchial) neuroendocrine tumors (LNETs) are relatively rare neoplasms, accounting for approximately 20–30% of all neuroendocrine tumors (NETs) and 1–2% of all lung malignancies in adults (1–3). LNETs represent the second most common primary site of NETs after the gastrointestinal (GI) tract. Their annual incidence ranges from 0.2 to 2.0 per 100,000 population, with a higher frequency in women (2, 4–6). However, the incidence of NETs, including LNETs, continues to rise. According to the Surveillance, Epidemiology, and End Results (SEER) Registry, the incidence of LNETs increased from 0.35 to 1.62 per 100,000 persons over a 39-year period (7). An increasing incidence has also been noted in younger patients, among whom the disease often follows a more aggressive course (8).

According to the 2022 World Health Organization (WHO) Classification of Endocrine and Neuroendocrine Neoplasm**s** (5th edition), LNETs are stratified based on mitotic activity into well-differentiated tumors (“lung carcinoids” (LC))—comprising typical carcinoid (TC) and atypical carcinoid (AC)—and poorly differentiated tumors, including large cell neuroendocrine carcinoma (LCNEC) and small cell lung carcinoma (SCLC) (9). In contrast to the gastroenteropancreatic (GEP) system, the 2022 WHO classification does not include the NET G1–G3 grading framework for pulmonary tumors, and the entity previously referred to as “NET G3” is no longer formally recognized in LNENs due to biological and morphological overlap with high-grade neuroendocrine carcinomas. Nevertheless, because several historical pathology reports and earlier clinical studies used a G1–G3 categorization in LNETs, we retained the term “LNET G3” in this manuscript only for the purpose of consistency with original diagnoses and comparability with previously published retrospective series. This approach aligns with recent expert commentary summarizing WHO 2022 updates for lung NENs (9). However, because the current WHO classification does not recognize a G3 NET category in lung NENs, the use of this terminology is non-standard, and any analyses involving this subgroup must be interpreted with extreme caution, without drawing generalizable conclusions.

LNETs constitute a heterogeneous group of tumors with variable clinical courses and prognoses depending on stage and grade at diagnosis. Current therapeutic options for unresectable or metastatic disease are limited and include somatostatin analogs (SSAs), the mammalian target of rapamycin (mTOR) inhibitor everolimus, chemotherapy (CHT), and radioligand therapy (RLT). However, robust randomized data to guide treatment sequencing are lacking, and international experts underscore the urgent need for further clinical trials. According to guidelines from the European Society of Medical Oncology (ESMO, 2021), the National Comprehensive Cancer Network (NCCN), and the European Neuroendocrine Tumor Society (ENETS), RLT is recommended as a preferred second-line option after SSA, or third-line after SSA and everolimus, in patients with somatostatin receptor (SSTR)–positive advanced disease, although no comparative studies of treatment strategies in LNETs have yet been conducted (10, 11).

The efficacy of RLT has been established primarily in GEP-NETs. In the NETTER-1 trial, [¹^77^Lu]Lu-DOTA-TATE significantly improved progression-free survival (PFS), overall response rate (ORR), and overall survival (OS) compared with high-dose octreotide in patients with progressive midgut NETs, while the NETTER-2 trial confirmed the benefit of RLT as first-line therapy in SSTR-positive, grade 2–3 GEP-NETs (12, 13). However, patients with LNETs were not included in these pivotal studies, and it remains uncertain whether their results can be extrapolated to this population. Similarly, no patients with LNETs were included in other phase III randomized clinical trials evaluating the efficacy of RLT, and current knowledge regarding its effectiveness and safety is derived exclusively from a limited number of mostly retrospective and case studies (14–32). Across these reports that provided data on tumor grade and administered isotope, which included between 6 and 48 patients with LNETs, treatment was generally well tolerated with an acceptable safety profile, and outcomes appeared at least partially comparable to those reported in the NETTER trials. It should be emphasized, however, that significant heterogeneity exists between studies in terms of treatment protocols, radionuclides used, and patient population characteristics. In seven studies assessing [¹^77^Lu]Lu-DOTA-TATE in LNETs, involving relatively small cohorts of 6–48 patients (with most series enriched for AC), median PFS ranged from 18 to 29 months and median OS from 37 to 59 months, with follow-up durations of up to 78 months (14–17, 19–22). Similarly, evidence regarding the efficacy of tandem RLT with [¹^77^Lu]Lu-DOTA-TATE/DOTA-TOC and [^90^Y]Y-DOTA-TATE/DOTA-TOC in LNETs is limited to only two small studies, including 4–37 patients overall, with about 20 individuals treated with true tandem protocols, reporting median PFS of 6–31 months and median OS of 40–61 months (17, 31).

The primary aim of this study was to evaluate the safety and efficacy of [¹^77^Lu]Lu-DOTA-TATE and tandem RLT with [^90^Y]Y-DOTA-TATE/[¹^77^Lu]Lu-DOTA-TATE in patients with LNETs, according to baseline clinical characteristics, SSTR expression, [¹^8^F]FDG PET/CT uptake, and prior antitumor therapies.

## 2 Materials and methods

This retrospective study included 22 patients with progressive LNETs who underwent RLT at the Department of Endocrinology and Radioisotope Therapy, Military Institute of Medicine – National Research Institute, Warsaw, Poland, between January 2013 and May 2025.

### 2.1 Eligibility criteria

Patients were eligible if they had histologically confirmed, disseminated, unresectable LNETs verified by core-needle biopsy or histopathological examination of surgical specimens. Additional requirements included radiologic evidence of disease progression (assessed according to RECIST version 1.1) or uncontrolled symptoms attributable to unresectable disease (33). Demonstration of SSTR expression in all measurable lesions was mandatory, assessed by either [^99m^Tc]Tc-HYNIC-TOC scintigraphy or [^68^;Ga]Ga-DOTA-0-Tyr³-Octreotate ([^68^;Ga]Ga-DOTA-TATE) PET/CT. SSTR uptake was considered adequate if it was equal to or greater than grade 2 on the modified Krenning Score (mKS), originally developed for [¹¹¹In]-octreotide scintigraphy and applied here as a semiquantitative method for both [^99m^Tc]Tc-HYNIC-TOC and [^68^;Ga]Ga-DOTA-TATE PET/CT. Scores ranged from 0 to 4, where 0 = no uptake; 1 = uptake lower than liver; 2 = uptake comparable to liver; 3 = uptake greater than liver but less than spleen; 4 = uptake greater than spleen.

In a subset of patients (n = 15), [¹^8^;F]FDG PET/CT was additionally performed, depending on clinical indications, primarily to exclude lesions lacking SSTR expression. FDG uptake was considered positive if greater than that of the liver. Because [¹^8^;F]FDG PET/CT was requested at the discretion of the treating physicians and performed in only 15 of 22 patients, FDG-related data are subject to information and selection bias, and all FDG-based analyses in this study should be regarded as exploratory.

All patients provided written informed consent for RLT in accordance with institutional protocols and ethical standards.

### 2.2 Follow-up and response assessment

After RLT completion, patients underwent follow-up imaging at 3 months and subsequently every 6 months. Anatomical imaging (CT or MRI, depending on baseline modality) was performed, and SSTR imaging was repeated annually using either [^68^;Ga]Ga-DOTA-TATE PET/CT or [^99m^Tc]Tc-HYNIC-TOC scintigraphy.

The first response evaluation was scheduled 3 months post-treatment and performed with CT or MRI. Responses were categorized as complete response (CR), partial response (PR), stable disease (SD), or progressive disease (PD) (**Supplementary Table A1**). Disease control rate (DCR) was defined as the sum of CR, PR, and SD. Long-term progression was determined according to RECIST 1.1.

PFS was defined as the time from initiation of RLT to documented disease progression, death from any cause, or last tumor evaluation. OS was defined as the time from initiation of RLT to death from any cause or last follow-up.

Hematologic, renal, and hepatic parameters were assessed prior to each course, at 2–4-week intervals between courses, and 2 weeks after the final RLT course. Adverse events were documented and graded according to CTCAE v5.0.

### 2.3 Study framework

This analysis reflects 13 years of single-center experience (September 2013–September 2025) and focused on PFS and OS. The study was conducted at a nationally designated ENETS Center of Excellence, which was the only institution in Poland offering uninterrupted RLT throughout the study period. Collaboration with national referral centers serving as regional hubs facilitated data collection and strengthened this analysis.

### 2.4 Treatment protocol

All patients received lutetium-based RLT, either as monotherapy (n = 14; 7.4 GBq [¹^77^Lu]Lu-DOTA-TATE; LutaPol^®^, Polatom, Otwock, Poland, or Lutathera^®^, Novartis) or as tandem therapy with lutetium and yttrium (n = 8; 1.85 GBq [^90^Y]Y-DOTA-TATE [ItraPol^®^, Polatom, Otwock, Poland] plus 1.85 GBq [¹^77^Lu]Lu-DOTA-TATE [LutaPol^®^]). The choice between monotherapy and tandem therapy was made by the multidisciplinary team on an individual, non-randomized basis, taking into account overall tumor burden, disease dynamics, and patient comorbidities. Courses were administered every 8–10 weeks, with post-therapy SPECT/CT performed after each course.

For clarity and consistency, the term RLT is used throughout this manuscript to refer to SSTR-targeted treatments with [¹^77^Lu]Lu-DOTA-TATE and [^90^Y]Y-DOTA-TATE, despite the fact that these treatments are also commonly referred to as peptide receptor radionuclide therapy (PRRT) in the literature.

Between courses, patients received long-acting somatostatin analogs (SSA) every 4 weeks—lanreotide (120 mg) or octreotide LAR (30 mg)—with a mandatory 4-week washout before each RLT course.

During hospitalization, all patients received standardized nephroprotection with amino acid (AA) infusions (Nephrotec^®^, Fresenius Kabi, Poland; 100 g/L). The regimen included 500 mL of AA before RLT, 500 mL during RLT (via a separate IV line), and 500 mL the following day. Additionally, patients received 1000 mL of balanced electrolyte solution on the treatment day and 500 mL the next day. Antiemetic prophylaxis with i.v. ondansetron (8–12 mg, adjusted to symptoms) was administered during AA infusion.

The planned four-course regimen was modified or discontinued in the event of adverse reactions, complications, or patient withdrawal of consent.

### 2.5 Laboratory evaluation

Fasting blood samples were collected between 7:30 and 8:30 a.m. at the Department of Endocrinology and Radioisotope Therapy, processed in the Department of Medical Diagnostics. Serum creatinine was measured enzymatically (Roche Diagnostics, Mannheim, Germany) using a COBAS c503 PRO analyzer (Hitachi High-Tech, Tokyo, Japan). eGFR was calculated with the CKD-EPI formula. Chromogranin A (CgA) was quantified by ELISA (Labor Diagnostika Nord, Nordhorn, Germany. Complete blood counts were analyzed with a Sysmex XN-1000 (Sysmex Corporation, Tokyo, Japan). Reference intervals are listed in **Supplementary Table A2**.

### 2.6 Statistical analysis

Analyses were performed using IBM SPSS Statistics (version 23; IBM Corp., Armonk, NY, USA) and the R statistical environment (version 4.3.1; R Core Team, 2023). The Shapiro–Wilk test was used to assess the normality of data distribution. Normally distributed variables are presented as mean ± standard deviation (SD), while non-normally distributed variables are summarized as medians (Med.) with interquartile ranges (IQR). Between-group comparisons were conducted using Student’s t-test or the Mann–Whitney U test, as appropriate, with equality of variances verified by Levene’s test. Pearson’s correlation coefficient was used to assess relationships between continuous variables. A two-tailed p-value < 0.05 was considered statistically significant. Only complete datasets were included in the analyses.

Progression-free survival (PFS) and overall survival (OS) were evaluated using Cox proportional hazards models. Variables with p < 0.10 in univariate analyses were subsequently included in multivariate models, with results reported as hazard ratios (HR) and 95% confidence intervals (CI). The proportional hazards assumption was verified using log-minus-log plots. The number of covariates in the multivariate models was limited due to the sample size. Given the very small cohort and low number of events, multivariable Cox models and all subgroup analyses were considered exploratory and at high risk of overfitting and both type I and type II errors; therefore, their results are interpreted descriptively and with caution.

## 3 Results

### 3.1 Patients baseline characteristics

A total of 22 patients with histologically confirmed LNETs were included in the final analysis (median age 61 years, 15 (68.2%) male) (**Supplementary Table A3**). Of these, 7 patients (31.8%) had TC, 12 patients (54.5%) had AC, and 3 patients (13.6%) had LNET G3. All cases were referred for treatment due to radiologically documented disease progression (n = 22 patients, 100.0%).

The median Ki-67 proliferation index for the entire cohort was 10% (range, 1–30%), whereas in the subgroup comprising only TC and AC the median Ki-67 was 7% (range, 1–20%). Metastatic disease was present in all patients, most frequently involving the liver (n = 15, 68.2%), bone (n = 17, 77.3%), and lymph nodes (n = 15, 68.2%). All individuals were receiving LSSA therapy at the time of RLT qualification - 72.7% were treated with lanreotide, and 27.3% with octreotide. Fourteen patients (63.6%) had undergone surgical resection of the primary tumor, 7 (31.8%) had previously received CHT, 8 (36.4%) had received radiotherapy to the primary tumor or metastatic sites, and 2 (9.1%) had a history of everolimus therapy. One patient had undergone two liver metastasis thermoablation procedures prior to RLT.

The majority of patients (54.5%) demonstrated high SSTR expression on baseline imaging, with a mKs of 4. Among the 15 patients who underwent [¹^8^;F]FDG PET/CT before treatment initiation, 10 (45.5%) exhibited at least one [¹^8^;F]FDG-avid lesion.

The baseline characteristics of the study population are summarized in **Table 1**.

**Table 1:** Baseline patients characteristics.

Baseline patients characteristics	
Parameter	Patients’ characteristics
Age, years median (IQR)	61 (52.7-68.7)
Sex:	
Male, n (%)	15 (68.2)
Female, n (%)	7 (31.8)
Grade:	
TC, n (%)	7 (31.8)
AC, n (%)	12 (54.5)
LNET G3, n (%)	3 (13.6)
Ki-67, median (IQR)	10.0 (2.0-20.0)
BMI, kg/m2, median (IQR)	25.6 (24.2-30.0)
Lymph nodes metastases	15 (68.2)
Distant metastases, n (%):	22 (100.0)
Liver metastases	15 (68.2)
Bone metastases	17 (77.3)
Other metastases	10 (45.4)
Obesity (BMI ≥30), n (%)	5 (22.7)
Carcinoid syndrome, n (%)	7 (31.8)
Prior treatment	
Surgery, n (%)	14 (63.6)
LSSA treatment:	22 (100)
Lanreotide, n (%)	16 (72.7)
Octreotide, n (%)	6 (27.3)
Chemotherapy, n (%)	7 (31.8)
Everolimus, n (%)	2 (9.1)
Radiotherapy, n (%)	8 (36.4)
Liver-directed therapy, n (%)	1 (4.5)
SSTR expression according to mKs	
Score 2, n (%)	5 (22.7)
Score 3, n (%)	5 (22.7)
Score 4, n (%)	12 (54.5)
Baseline [^18^F]FDG PE/CT expression	
<= liver, n (%)	5 (22.7)
>liver, n (%)	10 (45.5)
Not available, n (%)	7 (31.8)

AC, atypical carcinoid; BMI , body mass index, IQR, interquartile ranges, LNET - lung neuroendocrine tumor, LSSA – long-acting somatostatin analogue, mKs – modified Krenning score, TC – typical carcinoid.

### 3.2 Administered treatment

In the analyzed cohort, one patient received two courses of RLT (treatment was discontinued due to disease progression), one patient received three courses (treatment discontinued at the patient’s request for unclear reasons), and 20 patients completed the full four-course regimen. Thirteen patients received 4 × 7.4 GBq of [¹^77^Lu]Lu-DOTA-TATE, while seven patients were treated with tandem therapy consisting of 4 × 1.85 GBq of [^90^Y]Y-DOTA-TATE combined with 1.85 GBq of [¹^77^Lu]Lu-DOTA-TATE (**Table 2**).

**Table 2:** Cumulative administered activities in the study cohort.

Cumulative administered activities in the study cohort			
Type of therapy	Number of patients	Number of courses	Cumulative activity GBq
[¹^77^Lu]Lu-DOTA-TATE	14	All patients: 1 patient 2 courses 13 patients 4 courses	Median 29.6 14.8 29.6
Tandem therapy with [^90^Y]Y-DOTA-TATE/ [¹^77^Lu]Lu-DOTA-TATE	8	All patients: 1 patient 3 courses 7 patients 4 courses	Median 7.4/median 7.4 5.55/5.55 7.4/7.4

### 3.3 Primary response to RLT and long-term outcomes: evaluation of PFS and OS

During a median follow-up of 54.0 months (IQR, 14.5–66.0), 13 (59.1%) patients died, disease progression was documented in 19 (86.4%) patients, and 3 (13.6%) patients remained progression-free. Based on the anatomical assessment performed three months after completion of RLT according to RECIST 1.1 criteria, disease progression was observed in 2 (9.1%) patients, stable disease in 11 (68.2%) patients, partial response in 5 (22.7%) patients, while no complete responses were recorded (**Table 3**). A DCR of 90.9% was achieved. It should be noted that radiologic response was assessed 3 months after completion of RLT, which represents an early time point. Because RLT often produces delayed tumor shrinkage in well-differentiated NETs, early imaging may underestimate the full therapeutic effect. Thus, the 3-month response rates likely reflect early disease stabilization rather than the final extent of treatment benefit.

**Table 3:** Treatment response assessed 3 months after completion of RLT.

Treatment response assessed 3 months after completion of RLT	
Type of response	Number of patients
Complete response (CR), n (%)	0 (0.0)
Partial response (PR), n (%)	5 (22.7%)
Stable disease (SD), n (%)	15 (68.2%)
Progressive disease (PR), n (%)	2 (9.1%)

The median PFS and OS of the entire cohort were 16.0 months (95% CI, 11.2–20.8 months) and 62 months (95% CI, 30.7-93.3 months), respectively (**Table 4**). When restricting the analysis to patients with TC and AC, and with a median follow-up of 39 months (IQR, 16.2–72.5), the median PFS and OS for the entire cohort were 16 months (95% CI, 9.4–22.6) and 70 months (95% CI, 45.5–94.5), respectively.

**Table 4:** Median PFS and OS according to clinical and imaging subgroups, estimated using the Kaplan–Meier method and compared with the log-rank test.

Patients	Median PFS (months, 95% CI)	p (log-rank)	Median OS (months, 95% CI)	p (log-rank)
**All patients**	16.0 (11.2–20.8)	–	62.0 (30.7–93.3)	–
**Histology**		0.11		**0.015**
Typical carcinoid (TC)	25.0 (14.9–35.0)			
Atypical carcinoid (AC)	16.0 (13.9–18.0)		80.0 (42.5–117.5)	
LNET G3	14.0 (2.8–25.2)		34.0 (0.0–74.2)	
**SSTR uptake (SRI)** according to mKs		**0.021**		0.98
Uptake 4			70.0 (–)	
Uptake 2–3	34.0 (7.7–60.3)		62.0 (–)	
**FDG PET/CT uptake**		0.22		0.12
No uptake	40.0 (18.4–61.6)		134.0 (NE)	
FDG-avid	16.0 (2.6–29.4)		62.0 (8.0–116.0)	
**Treatment type**		**0.037**		0.054
[¹^77^Lu]Lu-DOTA-TATE monotherapy	16.0 (14.2–17.8)		34.0 (14.6–53.3)	
Tandem [¹^77^Lu]/[^90^Y] RLT	34.0 (0.6–67.4)		80.0 (54.3–105.6)	
**Prior chemotherapy**		0.22		**0.004**
No	40.0 (18.4-61.6)		80.0 (56.4–103.6)	
Yes	16.0 (2.6-29.4)		29.0 (7.3–50.7)	

NE, not estimable: fewer than 50% of patients experienced the event, so the median and its 95% CI could not be calculated.

“–” indicates that the 95% CI for the median could not be reliably estimated because of the low number of events.

CI, confidence interval; mKs, modified Krenning score; OS, overall survival; PFS, progression-free survival; SSTR, somatostatin receptor; SRI, somatostatin receptor imaging.

The bold values indicate p-values < 0.05, representing statistically significant results.

**Figure 1:**
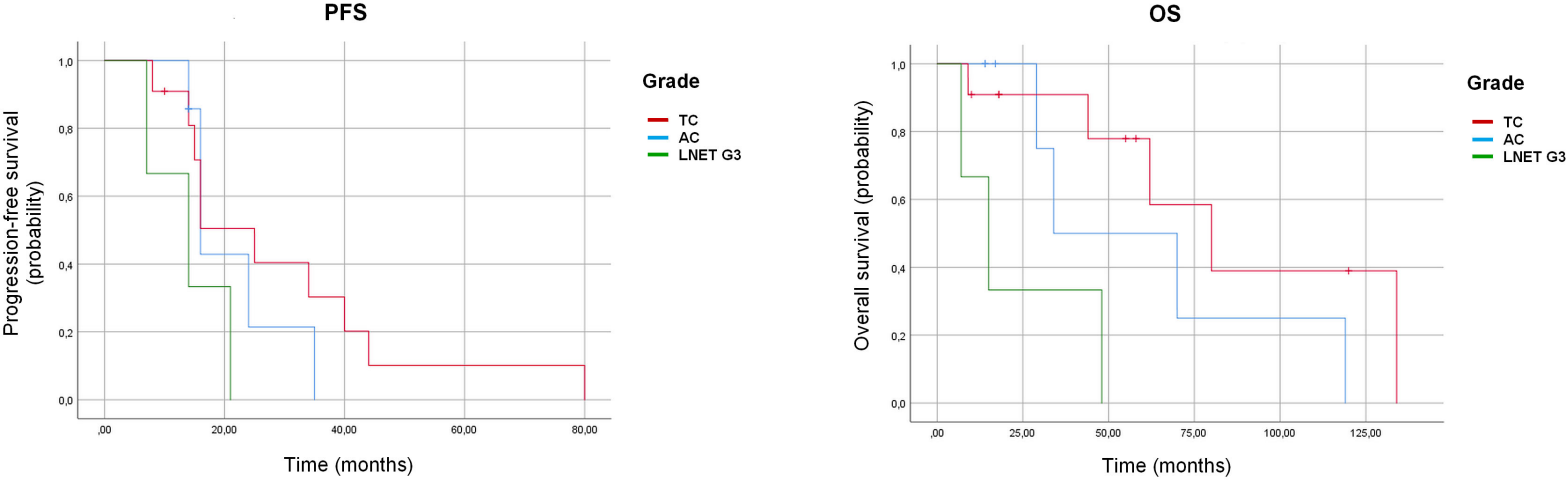
Kaplan–Meier curves for progression-free survival (PFS) left panel and overall survival (OS) right panel according to histological subtype. AC, atypical carcinoid; TC, typical carcinoid.

PFS in patients with TC, AC and LNET G3 was 25 months (95% CI: 14.9–35.0), 16 months (95% CI: 13.9–18.0), and 14 months (95% CI: 2.8–25.2), respectively, with no statistically significant difference between groups (p = 0.11). OS in the corresponding subgroups was 80 months (95% CI: 42.5–117.5), 34 months (95% CI: 0.0–74.2), and 15 months (95% CI: 2.2–27.8), showing a statistical significance (p = 0.015) (**Table 4**, **Figure 1**). No statistically significant correlation was found between Ki-67 index and PFS (p = 0.59) or OS (p = 0.99).

**Figure 2:**
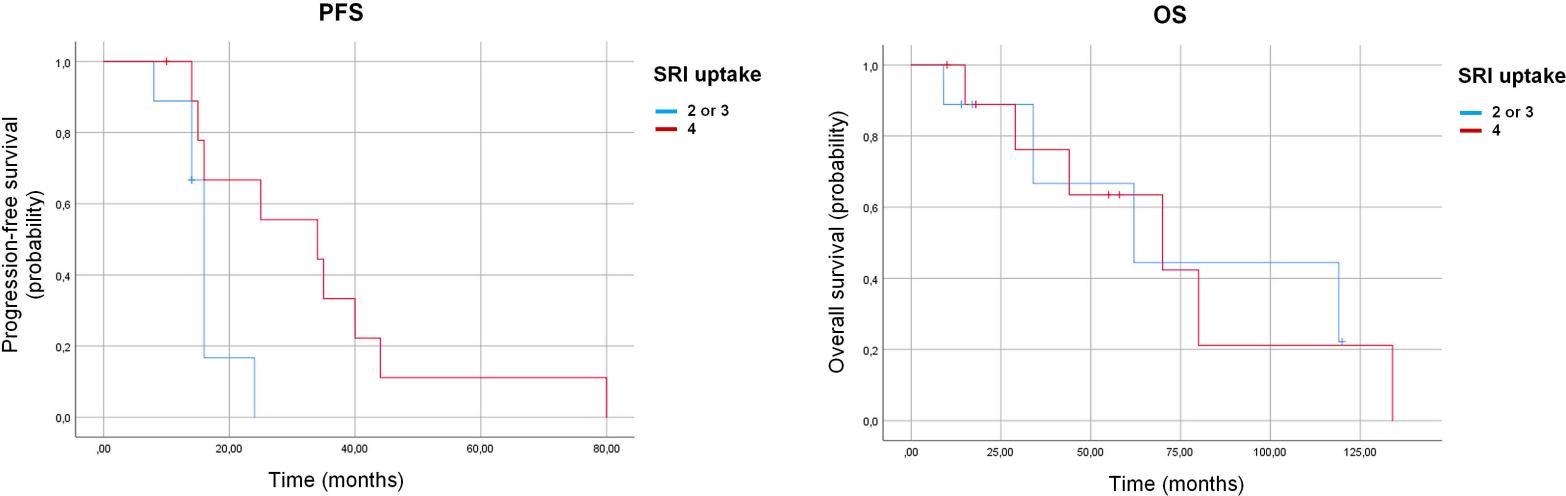
Kaplan–Meier curves for progression-free survival (PFS) left panel and overall survival (OS) right panel according to somatostatin receptor imaging (SRI) uptake: high uptake (grade 4 according to modified Krenning score) versus lower uptake (grades 2–3 according to modified Krenning score).

Patients with high SSTR uptake (grade 4 according to mKs) demonstrated significantly longer PFS compared with those with lower uptake (grades 2–3 according to mKs) (34.0 months, 95% CI: 7.7–60.3 vs. 16.0 months, 95% CI: 14.8–17.2; p=0.021) (**Table 4**, **Figure 2**). This difference remained statistically significant when restricting the analysis to patients with TC and AC only (p=0.018). In contrast, no significant difference in OS was observed between these subgroups (70 vs. 62 months; p=0.98). Because of the limited sample size, these subgroup findings may be prone to overinterpretation and should be viewed as exploratory.

A statistically significant correlation was observed between SSTR uptake and PFS (r = 0.47; p = 0.042), whereas no correlation was found between SSTR uptake and OS (p = 0.78).

A marked difference in both PFS and OS was observed between subgroups stratified by baseline [¹^8^;F]FDG PET/CT uptake in LNET lesions. Patients without [¹^8^;F]FDG-avid disease demonstrated a median PFS of 40 months (95% CI: 18.4–61.6) compared with 16 months (95% CI: 2.6–29.4) in those with FDG-avid tumors; however, this difference did not reach statistical significance (p=0.22). Similarly, median OS was 134 months (95% CI: not estimable) in patients without [¹^8^;F]FDG uptake versus 62 months (95% CI: 8.0–116.0) in patients with FDG-avid disease, which also failed to reach statistical significance (p=0.12) (**Table 4**). These analyses are based on a small subgroup with available [¹^8^;F]FDG PET/CT and therefore have limited statistical power. Given the limited and non-systematic use of [¹^8^;F]FDG PET/CT, these FDG-related comparisons should therefore be considered exploratory only and may be influenced by selection bias.

**Figure 3:**
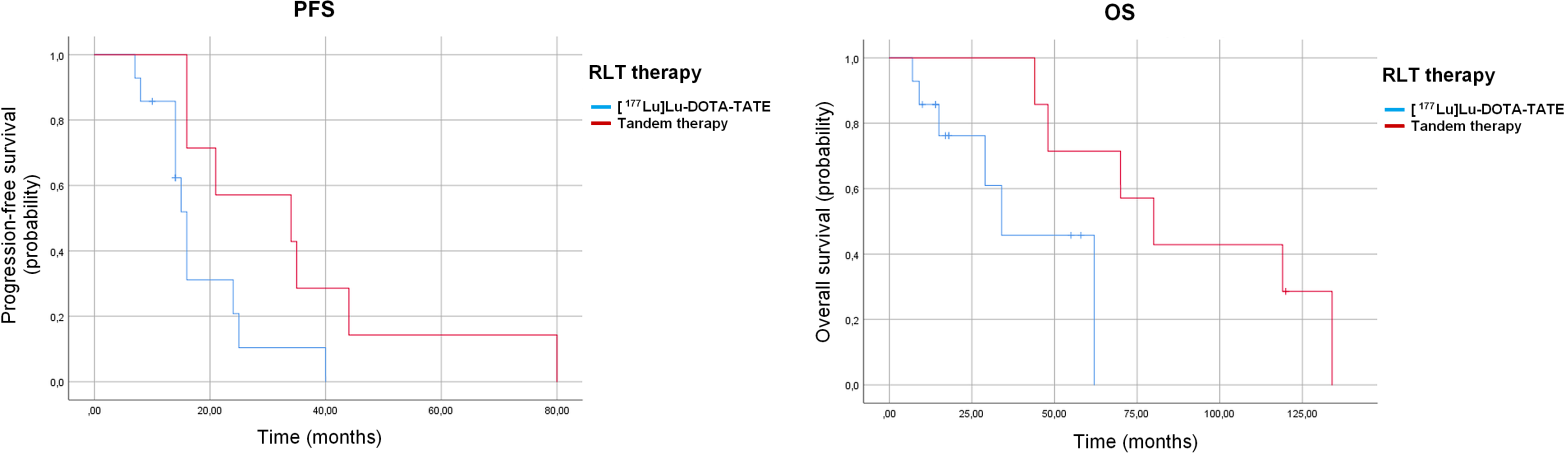
Kaplan–Meier curves for progression-free survival (PFS) left panel and overall survival (OS) right panel according to type of radioligand therapy: [¹^77^Lu]Lu-DOTA-TATE monotherapy versus tandem [¹^77^Lu]Lu-DOTA-TATE/[^90^Y]Y-DOTA-TATE therapy.

A statistically significant difference in PFS and a trend towards improved OS were observed between patients treated with [¹^77^Lu]Lu-DOTA-TATE monotherapy and those receiving tandem therapy with [¹^77^Lu]Lu-DOTA-TATE/[^90^Y]Y-DOTA-TATE in this small cohort. Median PFS was 16 months (95% CI: 14.2–17.8) in the monotherapy group versus 34 months (95% CI: 0.6–67.4) in the tandem group (p=0.037). Similarly, median OS was 34 months (95% CI: 14.6–53.3) for monotherapy compared with 80 months (95% CI: 54.3–105.6) for tandem therapy, showing a strong trend towards statistical significance (p=0.054) (**Table 4**, **Figure 3**). However, because the choice of treatment regimen was non-random and based on clinical judgement, these unadjusted comparisons are susceptible to treatment-selection bias and confounding by indication. Given the limited number of patients and events, these apparent differences should be interpreted cautiously and regarded as hypothesis-generating rather than as evidence of therapeutic superiority of tandem therapy.

A statistically significant difference in OS was observed according to prior exposure to CHT, with median OS reaching 80 months (95% CI: 56.4–103.6 months) in patients without a history of CHT compared to 29 months (95% CI: 7.3–50.7 months) in those previously treated (p = 0.004). This association should be interpreted cautiously, as CHT in clinical practice is usually reserved for patients with more advanced or aggressive disease and may therefore reflect confounding by indication rather than a detrimental effect of CHT itself. No statistically significant differences in either PFS or OS were observed according to prior history of primary tumor resection, radiotherapy, or everolimus treatment.

In the univariate Cox regression analysis of PFS, lower (2 or 3 according to mKs) SSTR uptake was significantly associated with a higher risk of progression (HR = 4.0, 95% CI: 1.0–16.3, p=0.049). Monotherapy compared with tandem therapy demonstrated a trend towards shorter PFS (HR = 0.3, 95% CI: 0.1–1.0, p=0.056). Other variables, including age, body mass index (BMI), Ki-67 index, [^18^F]FDG uptake, chromogranin A (CgA) concentration, prior treatments, and the presence of liver or bone metastases, were not significantly associated with PFS (**Table 5**). For OS, prior CHT was significantly associated with worse outcomes (HR = 0.1, 95% CI: 0.0–0.7, p=0.015). Tandem therapy was associated with a trend towards worse OS compared with monotherapy (HR = 4.6, 95% CI: 0.9–25.0, p=0.073), while higher BMI tended to correlate with longer OS (HR = 0.8, 95% CI: 0.6–1.0, p=0.10). Other factors, including age, Ki-67 index, SSTR uptake, [^18^F]FDG uptake, prior surgery, prior radiotherapy, prior everolimus, and the presence of liver or bone metastases, were not significantly associated with OS (**Table 5**).

**Table 5:** Univariate Cox regression analysis for progression-free survival (PFS) and overall survival (OS).

Variable	HR (95% CI), PFS	p (PFS)	HR (95% CI), OS	p (OS)
Age (continuous)	0.98 (0.9–1.0)	0.43	1.0 (0.9–1.1)	0.44
BMI (continuous)	0.9 (0.8–1.1)	0.42	0.8 (0.6–1.0)	0.10
Ki-67 (%)	1.0 (1.0–1.1)	0.29	1.0 (0.9–1.1)	0.45
Lower vs. high SSTR uptake according to mKs	4.0 (1.0–16.3)	**0.049**	1.0 (0.2–3.7)	0.98
FDG uptake (Yes vs. No)	2.2 (0.6–8.3)	0.24	0.2 (0.0–1.8)	0.15
Chromogranin A	1.0 (1.0–1.0)	0.84	1.0 (1.0-1.0)	0.71
Prior surgery (No vs Yes)	0.6 (0.2–1.7)	0.37	0.8 (0.2–3.2)	0.75
Liver metastases (No vs Yes)	1.0 (0.4–2.7)	1.00	0.4 (0.1–1.6)	0.21
Bone metastases (No vs Yes)	0.7 (0.2–2.0)	0.47	0.8 (0.2–2.9)	0.73
Prior chemotherapy (No vs Yes)	0.6 (0.2–1.5)	0.26	0.1 (0.0–0.7)	**0.015**
Prior radiotherapy (No vs Yes)	0.7 (0.2–1.9)	0.48	0.7 (0.2–2.4)	0.56
Prior everolimus (No vs Yes)	0.4 (0.1–1.9)	0.26	0.3 (0.0–3.2)	0.34
Monotherapy vs. tandem therapy	0.3 (0.1–1.0)	0.056	4.6 (0.9–25.0)	0.073

BMI, body mass index; mKs, modified Krenning score; SSTR, somatostatin receptor

The bold values indicate p-values < 0.05, representing statistically significant results.

In the multivariate Cox regression analysis for PFS, no independent prognostic factors reached statistical significance. Lower SSTR uptake (2 or 3 according to mKs) showed a strong trend towards an increased risk of progression (HR = 3.6, 95% CI: 0.9–14.7, p=0.069), while monotherapy compared with combination therapy also demonstrated a tendency towards inferior outcomes (HR = 2.8, 95% CI: 0.90–9.5, p=0.102). The Ki-67 index did not demonstrate prognostic relevance (HR = 1.0, 95% CI: 0.9–1.1, p=0.62).

For OS, none of the assessed variables retained statistical significance. Prior CHT was associated with a nonsignificant trend towards worse OS (HR = 4.3, 95% CI: 0.5–38.2, p=0.19). Similarly, monotherapy compared with tandem therapy was not independently predictive of OS (HR = 0.2, 95% CI: 0.0–2.7, p=0.22). SSTR uptake (HR = 0.8, 95% CI: 0.2–4.0, p=0.78) and Ki-67 index (HR = 1.0, 95% CI: 0.9–1.1, p=0.71) were not associated with survival outcomes. The absence of statistically significant predictors is most likely related to the limited sample size and the small number of events in this cohort, the observed associations in both univariate and multivariable models should be interpreted with great caution, as exploratory signals only, and not regarded as evidence of causal effects or of the comparative effectiveness of monotherapy versus tandem therapy or of prior chemotherapy exposure.

### 3.4 Complications of treatment

RLT was well tolerated, with an overall acceptable safety profile. The majority of treatment-related adverse events were grade 1–2 according to CTCAE 5.0. The most frequent toxicities included lymphocytopenia and anemia, occurring at comparable rates in both the [¹^77^Lu]Lu-DOTA-TATE and tandem therapy groups. In total, five grade 3 events were observed: four cases of lymphocytopenia and one case of thrombocytopenia. All of these events were transient, with hematological parameters returning to baseline values within 12 weeks after completion of therapy. There was no incidence of myelodysplasia/leukemia or renal toxicity on long-term follow-up. Data regarding treatment-related adverse events are summarized in **Table 6**.

**Table 6:** RLT toxicities in the whole cohort and in the treatment groups separately.

Toxicity	Grade	Overall (n=22)	[^177^Lu]Lu-DOTA-TATE group (n=13)	Tandem therapy group (n= 9)
Lymphopenia, n (%)	G0	9 (40.1)	3 (23.1)	6 (66.9)
	G1	8 (36.4)	7 (53.8)	1 (11.1)
	G2	1 (4.5)	0 (0.0)	1 (11.1)
	G3	4 (18.2)	2 (15.4)	2 (22.2)
Neutrocytopenia, n (%)	G0	21 (95.4)	12 (92.3)	9 (100.0)
	G1	1 (4.5)	1 (7.7)	0 (0.0)
Anemia, n (%)	G0	12 (54.5)	7 (53.8)	5 (55.5)
	G1	8 (36.4)	5 (38.5)	3 (33.3)
	G2	2 (9.1)	1 (7.7)	1 (11.1)
Thrombocytopenia, n (%)	G0	17 (77.3)	11 (84.6)	6 (66.7)
	G1	4 (18.2)	2 (15.4)	2 (22.2)
	G2	0 (0.0)	0 (0.0)	0 (0.0)
	G3	1 (4.5)	0 (0.0)	1 (11.1)
Serum creatinine increase, n (%)	G0	17 (77.3)	10 (76.9)	7 (77.8)
	G1	5 (22.7)	3 (23.1)	2 (22.2)
Transaminase increase, n (%)	G0	18 (81.8)	10 (76.9)	8 (88.9)
	G1	4 (18.2)	3 (23.1)	1 (11.1)

CI, confidence interval; mKs , modified Krenning score; NE, not estimable, OS, overall survival; PFS, progression-free survival; SRI, somatostatin receptor imaging; SSTR, somatostatin receptor. The severity of the adverse events was graded according to CTCAE version 5.0.

When comparing hematological parameters prior to the first treatment course and after the final course, a statistically significant decrease in neutrophil and lymphocyte counts, platelet levels, as well as hemoglobin concentration was observed. These data are presented in **Table 7**.

**Table 7:** Comparison of hematologic parameters, serum creatinine levels, and aspartate aminotransferase activity before and after last course of RLT.

Parameter	Before first RLT course Median [IQR]	After last RLT course Median [IQR]	p
Hemoglobin, g/dL	12.7 [11.0-13.6]	12.3 [11.2-13.9]	**0.002**
Blood platelets, x109/l	292.0 [238.0 – 334.0]	252.0 [205.0-301.0]	**0.003**
Neutrophils, x103/µL	4.0 [3.2-5.0]	2.9 [2.3-4.5]	0.051
Lymphocytes, x103/µL	1.6 [1.3-2.0]	1.0 [0.6-1.1]	**<0.001**
Creatinine, mg/dl	0.9 [0.8-1.1]	0.9 [0.8-1.2]	0.24
AST, U/l	29.0 [21.0-39.0]	27.0 [19.0-37.0]	0.37

AST, aspartate aminotransferase, GFR , glomerular filtration rate, IQR, interquartile ranges; RLT , radioligand treatment

The bold values indicate p-values < 0.05, representing statistically significant results.

When comparing hematologic and biochemical parameter changes between patients treated with [¹^77^Lu]Lu-DOTA-TATE monotherapy and those receiving tandem therapy, no statistically significant differences were observed in the decline of platelet count, hemoglobin concentration, total white blood cell count, neutrophils, creatinine, or AST activity (all p > 0.10, Mann–Whitney U test). A trend towards a greater decrease in lymphocyte count was noted in the tandem therapy group compared with monotherapy (p=0.096). Prior history of CHT was not associated with increased treatment-related toxicity (all p > 0.10, Mann–Whitney U test).

## 4 Discussion

Despite more than 30 years of global experience with RLT, evidence specific to LNETs remains scarce, with available studies characterized by small patient cohorts and frequently incomplete data (14–31). A major challenge in assessing the true effectiveness of RLT is the heterogeneity of treatment protocols, including variability in administered doses and the concurrent use of radiosensitizing CHT in some studies.

In our cohort, RLT achieved a disease control rate of 90.9%, with no CR, 22.7% PR, and the majority of patients (68.2%) maintaining SD three months after treatment. Median PFS and OS for the entire population were 16.0 and 62 months, respectively, while in the subgroup of TC and AC patients, outcomes were slightly more favorable (PFS 16 months, OS 70 months). When stratified by histology, patients with TC showed the longest PFS and OS (25 and 80 months), followed by AC (16 and 34 months), whereas those with LNET G3 had the poorest outcomes (14 and 15 months). Importantly, OS differed significantly between subgroups (p = 0.015), while PFS did not (p = 0.11), and no association between Ki-67 index and survival was observed.

The achieved median PFS and OS in our study were somewhat shorter than outcomes typically reported for GEP-NETs treated with RLT. In NETTER-1, median PFS reached ~25 months in the [¹^77^Lu]Lu-DOTA-TATE arm, and the COMPETE trial similarly reported a median PFS of 23.9 months with [¹^77^Lu]Lu-edotreotide (12, 33–35). By contrast, retrospective LNET series have shown median PFS of 18–27 months and OS of 40–59 months, which is consistent with our findings and with the generally less favorable prognosis more aggressive disease biology of LNETs (14–16). Accordingly, any numerical comparison with GEP-NET trials (NETTER-1, NETTER-2, COMPETE) should be viewed as purely descriptive, as substantial differences in trial design, inclusion criteria and underlying tumor biology preclude interpreting these data as evidence of comparable or inferior efficacy of RLT in LNETs.

Although in our cohort the Ki-67 proliferation index was not associated with PFS or OS after RLT, this likely reflects the very small sample size and limited number of events. In larger digestive NET series, Ki-67 remains prognostically relevant: Massironi et al. demonstrated that a 10% cutoff identifies biologically distinct subgroups, with Ki-67 ≥10% associated with markedly shorter PFS and worse outcomes (36). Thus, our findings should not be interpreted as contradicting the prognostic value of Ki-67 but rather as indicating that, in this small LNET cohort, Ki-67 did not emerge as a predictor of response to RLT.

A unique aspect of our study is the inclusion of LNET G3 cases with preserved SSTR expression. Although our small cohort suggests that RLT may provide disease stabilization in selected LNET G3 patients, the subgroup is extremely limited and the G3 designation is non-standard in current WHO lung classifications. Therefore, these observations cannot be generalized and should be regarded as exploratory, with a high risk of overinterpretation. Given that only three patients in our cohort were classified as LNET G3, the results are too limited to draw any conclusions regarding prognosis or treatment efficacy in this population.

In our cohort, tandem therapy was associated in unadjusted analyses with numerically longer PFS and OS than [¹^77^Lu]Lu-DOTA-TATE monotherapy (median PFS 34 vs 16 months, p=0.037; OS 80 vs 34 months, p=0.054), and Kaplan–Meier curves demonstrated the same pattern. Cox regression similarly suggested a trend toward shorter PFS with monotherapy (HR = 0.3, 95% CI 0.1–1.0), although the multivariable model yielded non-significant and directionally unstable estimates for OS (HR = 4.6, 95% CI 0.9–25.0), reflecting the limited number of events and wide confidence intervals. These findings align with prior NET reports indicating potential benefit of tandem therapy (17, 37, 38), and the complementary radiophysical properties of ¹^77^Lu and ^90^Y offer a plausible mechanistic rationale; however, in our dataset this remains speculative. Crucially, treatment allocation was non-random and based on clinical judgement, making these unadjusted comparisons prone to treatment-selection bias and confounding by indication. Therefore, the apparent advantage of tandem therapy should be regarded as hypothesis-generating only and requires confirmation in prospective, adequately powered, ideally randomized studies.

In our cohort, univariate analysis revealed that lower SSTR uptake was associated with shorter PFS. Previous studies have consistently shown that high SSTR uptake on SRI is a robust prognostic factor for favorable response to RLT in patients with NETs (15, 39–41). Similar results have also been reported in cohorts of LNETs, where higher SRI uptake correlated with improved treatment outcomes (42). In agreement with these findings, our data support the notion that SRI uptake is an important predictor of therapeutic efficacy in LNET in this cohort, although the small sample size precludes firm conclusions about its prognostic strength relative to histological grading.

In our cohort, prior CHT was associated with worse OS in the univariate analysis, but this effect did not persist in the multivariate model. This pattern likely reflects confounding by indication, as CHT in LNETs is typically administered to patients with more aggressive disease, who also tend to present with adverse features such as higher Ki-67, FDG-avid lesions, or lower SSTR expression. Once these covariates were accounted for, the apparent prognostic impact of CHT diminished. The small sample size and limited number of events further constrain the statistical power of multivariable modelling. Overall, these findings are consistent with the interpretation that prior CHT functions primarily as a surrogate marker of unfavorable tumor biology rather than an independent determinant of survival, although this remains speculative given the retrospective design and limited number of events.

The prognostic value of FDG uptake has been consistently demonstrated in larger NET cohorts (43–45). In our study, although notable differences in PFS and OS were observed across [¹^8^;F]FDG PET/CT subgroups, these comparisons did not reach statistical significance. This lack of significance should be interpreted cautiously, as the limited sample size reduces statistical power and does not exclude a clinically relevant effect. Furthermore, FDG PET/CT was performed in only 15/22 patients based on clinical judgement and may have been preferentially obtained in those with lower SSTR uptake, higher Ki-67, or other features indicative of more aggressive disease. Such potential selection bias further limits the generalizability of our findings and reinforces their exploratory nature.

In the context of available systemic options for advanced LNETs, our RLT outcomes compare favorably with published series and appear promising relative to alternative therapies. SSA, typically used as first-line treatment in slowly progressive, SSTR-positive disease, provide meaningful disease control; in SPINET, lanreotide achieved a median PFS of 16.6 months, with OS not reached (46). Similarly, in a retrospective single-center series of metastatic LNETs treated with first-line SSA, Bongiovanni et al. reported a median PFS of 11.1 months, (with longer PFS in [¹^8^;F]FDG PET/CT-negative compared with FDG-positive tumors (15.2 vs. 7.0 months)) (47). For everolimus—the only approved targeted therapy for LNETs—efficacy has been modest: in the RADIANT-4 lung subgroup, PFS was 9.2, and in the LUNA study ~33% of patients remained progression-free at 9 months, with objective responses being rare (48, 49). Although some mixed-NET retrospective cohorts (dominated by GEP-NETs) reported PFS up to ~29 months, these findings are difficult to extrapolate to LNETs (50). In parallel, emerging expert commentaries have emphasized the biological rationale for combining SSA with mTOR inhibitors or with RLT, highlighting potential synergistic interactions and strategies to overcome or delay SSA resistance (51). Supporting the relevance of treatment sequencing, data from a large multicenter real-world cohort by Pusceddu et al. suggest that earlier use of RLT—administered immediately after progression on SSA—was associated with significantly longer PFS compared with upfront chemotherapy or targeted therapy in well-differentiated GEP-NETs, underscoring the importance of timing in SSTR-positive disease (52). Notably, even patients with lower SRI uptake in our cohort (mKs 2–3) experienced PFS (16.0 months) and OS (62 months) that compare favorably with everolimus benchmarks. Importantly, however, the applicability of RLT is inherently restricted to patients with sufficient SSTR expression, underscoring the biological prerequisites that guide treatment selection.

The role of CHT in LNETs remains limited and is strongly dependent on histology and tumor grade (53–56). In TC, cytotoxic regimens have very low efficacy, with rare objective responses and PFS generally inferior to SSA or targeted therapies (57). In AC, CHT shows somewhat greater but still modest activity, with limited durability (58). Temozolomide-based CAPTEM yields ORR around 18% and median PFS 9–13 months, with OS ranging from 30 to 68 months across small series (56–58). Platinum–etoposide regimens in TC/AC achieve ORR of 23–39% but with short PFS (~7 months) and limited OS (59–61). In well- and moderately differentiated thoracic NETs (including LNET), the phase II ATLANT trial indicated that lanreotide plus temozolomide may provide clinical benefit with manageable toxicity, supporting the consideration of combination strategies in selected patients with progressive disease (62). In high-grade LNETs, particularly LCNEC, platinum-based therapy remains standard, producing ORR ~40–45% but with short durability (median PFS 4–6 months, OS 8–15 months) (63–65). Overall, these findings indicate that CHT is best reserved for high-grade or rapidly progressive disease, as its role in TC and AC is marginal compared with receptor-directed or targeted treatments.

In our cohort, RLT was well tolerated, with mainly mild to moderate, transient hematologic toxicity and no long-term renal or hepatic events, in line with previous GEP-NET and LNET series (66, 67). In the NETTER-1 trial, [¹^77^Lu]Lu-DOTA-TATE was likewise associated predominantly with low-grade hematologic adverse events and very infrequent clinically significant renal toxicity, supporting its overall favorable safety profile (38). Consistent with these observations, the few available RLT studies in lung NETs have also reported mostly mild toxicity, with grade 3 events occurring only sporadically (16, 17, 20, 21, 30).

Available data indicate that tandem RLT with ¹^77^Lu- and ^90^Y-labelled SSAs has a safety profile broadly comparable to [¹^77^Lu]Lu-DOTA-TATE monotherapy, with long-term series reporting high-grade hematologic or renal events only rarely (37, 42, 68). Mariniello et al. nevertheless identified combined ¹^77^Lu/^90^Y treatment as an independent risk factor for moderate-to-severe leukopenia in LNETs, underscoring the need for careful hematologic monitoring. In our cohort, a trend towards a greater lymphocyte decline was observed in the tandem group, but overall toxicity did not differ meaningfully between regimens, and prior CHT was not associated with increased adverse events. Overall, these findings support RLT—including tandem protocols—as a generally well-tolerated option in LNETs, with predominantly hematologic but usually manageable toxicity.

## 5 Study limitations

The present study has several limitations that should be acknowledged. The most important one is the relatively small sample size, however, is comparable to that of previously published reports evaluating the efficacy and safety of RLT in LNETs. This limitation is largely related to the restricted access to RLT in this patient population and the lack of clear consensus on the optimal timing of treatment initiation in current guidelines. Another limitation is the retrospective design, which resulted in incomplete availability of some follow-up data, particularly laboratory tests beyond the early post-treatment period, precluding a comprehensive evaluation of long-term AE. Furthermore, the single-center nature of the study may limit the generalizability of our findings, and the heterogeneity of the cohort in terms of prior therapies and histological subtypes should also be considered when interpreting the results. In addition, allocation to [¹^77^Lu]Lu-DOTA-TATE monotherapy versus tandem [¹^77^Lu]Lu-DOTA-TATE/[^90^Y]Y-DOTA-TATE, and the decision to administer prior CHT, were made on an individual, non-randomized basis, which introduces substantial risk of treatment-selection bias and confounding by indication. Finally, although we performed both univariate and multivariate analyses, the limited number of events reduces the statistical power to identify independent prognostic factors with certainty. In particular, the subgroup comparisons and multivariable Cox models are underpowered and at high risk of overfitting; therefore, the observed associations may represent chance findings and are susceptible to both false-positive and false-negative results. These analyses should therefore be regarded as exploratory and interpreted with great caution, without implying causal relationships or therapeutic superiority of one regimen over another.

Despite these limitations, our work adds valuable evidence to the very limited literature on RLT in LNETs, supported by long-term follow-up in an ENETS-accredited center and a detailed subgroup analysis by tumor grade, including, for the first time, patients with LNET G3 and high SSTR expression.

## 6 Conclusions

Our study provides one of the most comprehensive analyses to date of RLT in LNETs, a population for whom high-quality evidence remains extremely limited. We confirmed that RLT is both active and safe, achieving clinically meaningful PFS and OS outcomes in long-term follow-up, even in advanced disease, and that exploratory subgroup analyses suggest more favorable outcomes in patients with strong SSTR expression and in those receiving tandem therapy. Importantly, our findings extend existing knowledge by including a unique subgroup of LNET G3 patients with preserved SSTR expression, for whom treatment options are particularly scarce. Collectively, these exploratory observations are consistent with a potential role of RLT as a valuable therapeutic strategy in LNETs. However, because current evidence — including our own — is based on small, retrospective series with exploratory subgroup and multivariable analyses, the observed differences between monotherapy and tandem therapy must be considered hypothesis-generating only, and no firm conclusions regarding comparative efficacy can be drawn. These limitations further support the need for prospective, randomized studies to define the optimal use of RLT in LNETs, analogous to established evidence in GEP-NETs. Findings related to the small subgroup historically labeled as LNET G3 are exploratory only and cannot be generalized to high-grade pulmonary neuroendocrine neoplasms.

## Data Availability

The original contributions presented in the study are included in the article/**Supplementary Material**. Further inquiries can be directed to the corresponding author.
