# Supplementary figures and images for: The impact of radioligand therapy on prognosis in patients with lung neuroendocrine tumors
Source: Front Endocrinol (Lausanne). 2026 Jan 26;17:1738286. doi: 10.3389/fendo.2026.1738286 (PMC12883361; doi:10.3389/fendo.2026.1738286)

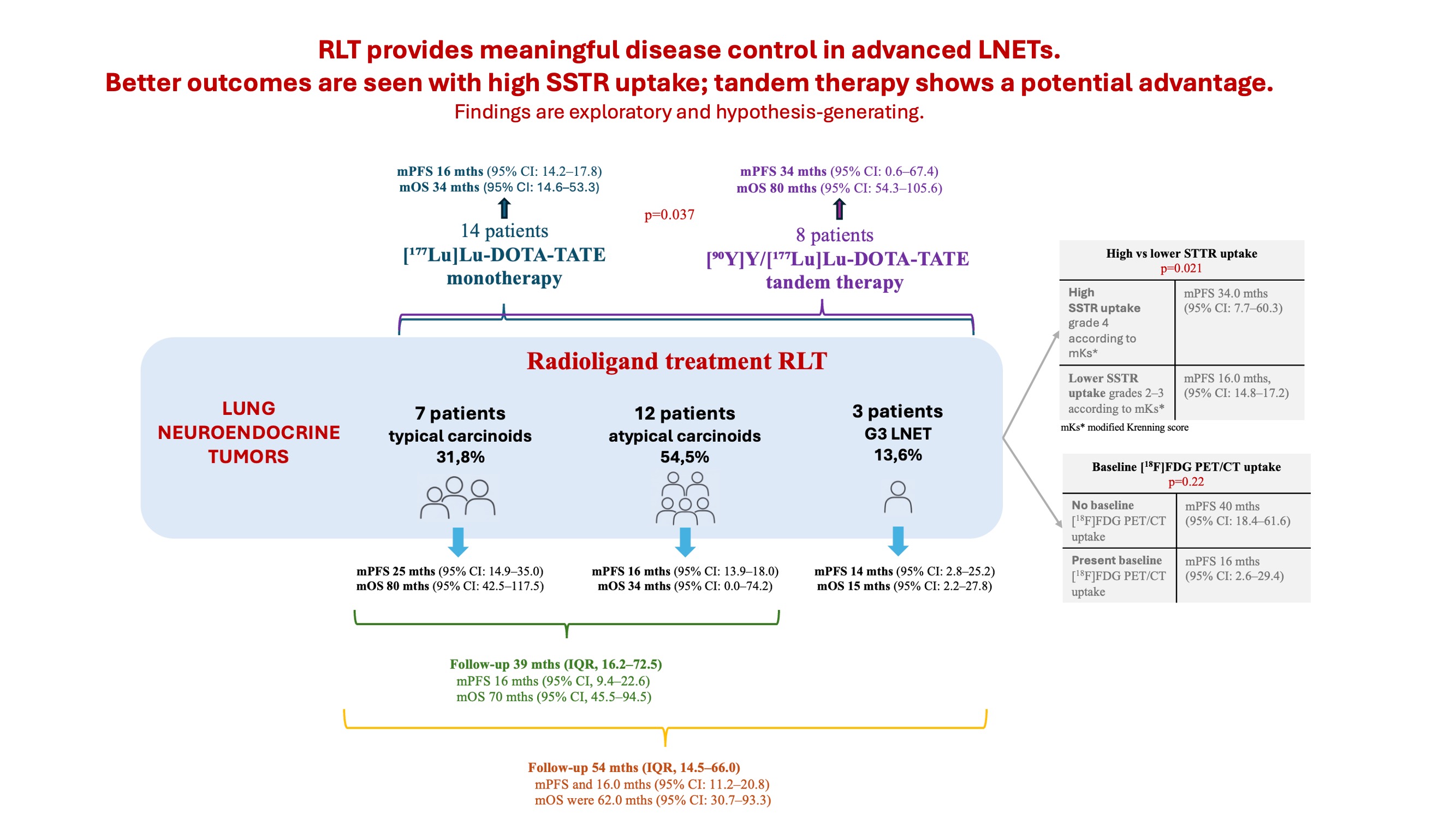

Supplement: Supplementary file 1 [file Image1.jpeg]
