# Supplementary material for: The impact of radioligand therapy on prognosis in patients with lung neuroendocrine tumors
Source: Front Endocrinol (Lausanne). 2026 Jan 26;17:1738286. doi: 10.3389/fendo.2026.1738286 (PMC12883361; doi:10.3389/fendo.2026.1738286)
Supplement: Supplementary file 2 [file Table1.docx]

**Supplementary Table A1**. Response categories according to RECIST 1.1

| **Response category** | **Definition** |
| --- | --- |
| Complete Response (CR) | Disappearance of all target lesions. All pathological lymph nodes must have decreased to <10 mm in short axis. |
| Partial Response (PR) | At least a 30% decrease in the sum of diameters of target lesions, taking baseline sum diameters as reference. |
| Stable Disease (SD) | Neither sufficient shrinkage to qualify for PR nor sufficient increase to qualify for PD, taking the smallest sum diameters (nadir) as reference. |
| Progressive Disease (PD) | At least a 20% increase in the sum of diameters of target lesions, taking as reference the smallest sum on study (nadir), with an absolute increase of at least 5 mm, **or** appearance of new lesions, **or** unequivocal progression of non-target lesions. |

**Supplementary Table A2**. Reference Ranges for Biochemical and Hematological Parameters

| **Parameter** | **Unit** | **Reference range** |
| --- | --- | --- |
| Creatinine | mg/dL | 0.7 - 1.2 |
| GFR | mL/min/1.73 m² | >60 |
| AST | U/l | 1-18 |
| Leukocytes | x109/L | 4.30 - 9.64 |
| Erythrocytes | x1012/L | 4.36 - 5.78 |
| Blood platelets | x109/L | 163 - 347 |
| Neutrophils | x103/µL | 1.93 - 5.87 |
| Lymphocytes | x103/µL | 1.23 - 3.42 |
| Chromogranin A | ng/mL | <100 |

AST - aspartate aminotransferase, GFR – glomerular filtration rate

**Supplementary Table A3**. Patients’ characteristics

| **Nr** | **Sex** | **Age** | **Grade** | **Ki-67 index** | **Prior LNET primary lesion surgery** | **Other treatment prior to RLT** | **Disease extent prior to RLT** | **Indications for RLT** | **SRI uptake** | **[18F]FDG**  **PET/CT uptake** |
| --- | --- | --- | --- | --- | --- | --- | --- | --- | --- | --- |
| 1 | M | 61 | TC | 1 | 1 | SSA | Local recurrence of the primary tumor; pleura, liver, lymph nodes, bones metastases | Enlargement of local recurrence; new metastatic lesions in lymph nodes | 3 | 0 |
| 2 | M | 72 | AC | 7 | 1 | SSA;  radiotherapy of local recurrence; chemotherapy: PE regimen;  chemotherapy: Paclitaxel + carboplatin | Local recurrence of the primary tumor; lungs, pleura, lymph nodes metastases | Enlargement of local recurrence; new metastatic lesions in lungs and lymph nodes | 2 | 1 |
| 3 | M | 55 | AC, | 5 | 0 | SSA | Primary lung lesion; lymph nodes, bone metastases. | Increase in size of primary lesion; new metastatic lymph node lesions | 2 | 1 |
| 4 | M | 73 | LNET G3 | 30 | 1 | SSA;  chemotherapy: CAPTEM regimen;  radiotherapy of bone and liver metastases | Liver, spleen, bone metastases | Increase in number and size of liver, bone, and splen metastases | 4 | 0 |
| 5 | M | 42 | TC | 1 | 0 | SSA;  everolimus | Primary lung lesion; lymph nodes, liver and bone metastases | Increase in size of primary lesion; increase in number and size of liver, bone and lymph node metastases | 3 | 1 |
| 6 | F | 66 | TC | 2 | 0 | SSA;  chemotherapy: CAPTEM regimen | Primary lung lesion; liver and bone metastases | New liver and bone lesions | 2 | 1 |
| 7 | M | 65 | AC | 15 | 0 | SSA;  radiotherapy of the primary lung lesion | Primary lung lesion; lymph nodes, lung, liver and bone metastases | New liver and bone lesions | 4 | - |
| 8 | M | 46 | AC | 4 | 1 | SSA;  radiotherapy of bone metastases | Lymph nodes, bone metastases | Increase in number and size of bone metastases | 4 | - |
| 9 | M | 62 | AC | 20 | 0 | SSA | Primary lung lesion; liver, spleen and bone metastases | Increase in size of primary lesion; increase in number and size of liver metastases | 4 | 1 |
| 10 | F | 52 | TC | 2 | 0 | SSA | Primary lung lesion; lung and lymph nodes metastases | Increase in size of primary lesion; increase in number and size of lymph node metastases | 4 | 1 |
| 11 | M | 55 | AC | 10 | 1 | SSA;  radiotherapy of bone metastases | Primary lung lesion; lymph nodes and bone metastases | Increase in number and size of bone metastases | 4 | - |
| 12 | M | 49 | LNET G3 | 25 | 0 | SSA;  radiotherapy of primary lesion and bone metastases; chemotherapy: docetaxel;  chemotherapy: CAPTEM regimen | Primary lung lesion; lymph nodes, liver and bone metastases | New liver metastases; increase in number of bone metastases | 3 | 1 |
| 13 | F | 72 | AC | 10 | 1 | SSA;  Chemotherapy: cisplatin + vinorelbine | Local recurrence of the primary tumor; pleura, lymph nodes, liver and bone metastases | Enlargement of local recurrence; new metastatic lesions in bones; increase in size of liver metastases | 4 | 0 |
| 14 | M | 56 | AC | 10 | 1 | SSA | Lymph nodes, lung, liver, and bone metastases | New liver metastases; increase in number of lymph nodes and bone metastases | 4 | - |
| 15 | F | 73 | AC | 20 | 1 | SSA | Lung and bone metastases | Increase in number and size of bone metastases | 2 | 1 |
| 16 | M | 32 | TC | 2 | 1 | SSA;  Chemotherapy: PE regimen;  everolimus | Lymph nodes, liver, and bone metastases | Increase in number and size of bone metastases | 4 | - |
| 17 | M | 71 | TC | 2 | 1 | SSA | Local recurrence of the primary tumor; lymph nodes, liver, right adrenal gland and bone metastases | Increase in the size of liver, adrenal, and bone metastases | 3 | 1 |
| 18 | F | 56 | TC | 2 | 1 | SSA;  radiotherapy of primary lesion | Lymph nodes and liver metastases | Increase in the size of liver metastases | 3 | - |
| 19 | M | 68 | AC | 10 | 1 | SSA | Liver, and bone metastases | Increase in number and size of liver and bone metastases | 4 | 1 |
| 20 | F | 67 | LNET G3 | 25 | 0 | SSA;  Chemotherapy: PE regimen;  radiotherapy of the primary lung lesion | Primary lung lesion; liver metastases | Increase in number and size of liver metastases | 4 | 1 |
| 21 | M | 65 | AC | 8 | 1 | SSA;  two liver metastasis thermoablations | Lymph nodes, pleura, liver, and bone metastases | New liver metastases | 4 | - |
| 22 | F | 53 | AC | 20 | 1 | SSA | Lung, lymph nodes and pleura metastases | New pleura metastases | 2 | 0 |

AC- atypical carcinoid, CAPTEM regimen: capecitabine + temozolomide, F-female, LNET lung neuroendocrine tumor, M- male, PE regimen: cisplatin + etoposide, RLT- radioligand treatment, SRI -Somatostatin receptor imaging, SSA- somatostatin analogue, TC- typical carcinoid.
